# MRI-based visualization of rTMS-induced cortical plasticity in the primary motor cortex

**DOI:** 10.1371/journal.pone.0224175

**Published:** 2019-10-24

**Authors:** Kaori Tamura, Takahiro Osada, Akitoshi Ogawa, Masaki Tanaka, Akimitsu Suda, Yasushi Shimo, Nobutaka Hattori, Koji Kamagata, Masaaki Hori, Shigeki Aoki, Takahiro Shimizu, Hiroyuki Enomoto, Ritsuko Hanajima, Yoshikazu Ugawa, Seiki Konishi

**Affiliations:** 1 Department of Neurophysiology, Juntendo University School of Medicine, Tokyo, Japan; 2 Department of Neurology, Juntendo University School of Medicine, Tokyo, Japan; 3 Department of Radiology, Juntendo University School of Medicine, Tokyo, Japan; 4 Department of Neurology, Tottori University School of Medicine, Tottori, Japan; 5 Department of Neuro-Regeneration, Fukushima Medical University, Fukushima, Japan; 6 Research Institute for Diseases of Old Age, Juntendo University School of Medicine, Tokyo, Japan; 7 Sportology Center, Juntendo University School of Medicine, Tokyo, Japan; 8 Advanced Research Institute for Health Science, Juntendo University School of Medicine, Tokyo, Japan; University of Ontario Institute of Technology, CANADA

## Abstract

Repetitive transcranial magnetic stimulation (rTMS) induces changes in cortical excitability for minutes to hours after the end of intervention. However, it has not been precisely determined to what extent cortical plasticity prevails spatially in the cortex. Recent studies have shown that rTMS induces changes in “interhemispheric” functional connectivity, the resting-state functional connectivity between the stimulated region and the symmetrically corresponding region in the contralateral hemisphere. In the present study, quadripulse stimulation (QPS) was applied to the index finger representation in the left primary motor cortex (M1), while the position of the stimulation coil was constantly monitored by an online navigator. After QPS application, resting-state functional magnetic resonance imaging was performed, and the interhemispheric functional connectivity was compared with that before QPS. A cluster of connectivity changes was observed in the stimulated region in the central sulcus. The cluster was spatially extended approximately 10 mm from the center [half width at half maximum (HWHM): approximately 3 mm] and was extended approximately 20 mm long in depth (HWHM: approximately 7 mm). A localizer scan of the index finger motion confirmed that the cluster of interhemispheric connectivity changes overlapped spatially with the activation related to the index finger motion. These results indicate that cortical plasticity in M1 induced by rTMS was relatively restricted in space and suggest that rTMS can reveal functional dissociation associated with adjacent small areas by inducing neural plasticity in restricted cortical regions.

## Introduction

Transcranial magnetic stimulation (TMS) is a non-invasive method to induce neural activity of stimulated regions or block their functions transiently and is also capable of changing behavior [1–4]. Since behavioral changes are thought to result primarily from changes in neural activity in the stimulated region and connectivity with other brain regions, it is important to understand the spatial extent of the effect of stimulation that prevails in the stimulated region. Electric field measurements have provided the spatial distribution of field strength in the stimulated region [5–12]. Concurrent measurements using functional magnetic resonance imaging (fMRI) and TMS have also revealed the spatial distribution of MRI signals in local and remote brain regions elicited by magnetic stimulation [13–18]. Repetitive TMS (rTMS), on the other hand, has been used to induce changes in cortical excitability of stimulated regions for minutes to hours after the end of the intervention, which may result in behavioral changes [1–4]. It is also important to understand the spatial extent of cortical plasticity induced by rTMS. However, the visualization of the spatial extent of cortical plasticity remains largely uninvestigated.

Recent MRI studies have investigated the effects of rTMS on functional connectivity and revealed changes in functional connectivity between the stimulated region and other brain regions after rTMS [19–44]. Our previous study has demonstrated changes in “interhemispheric” functional connectivity, the resting-state functional connectivity between the stimulated region and the symmetrically corresponding region in the contralateral hemisphere [33]. It found that, after stimulation to the left primary motor cortex (M1), inhibitory rTMS increased interhemispheric functional connectivity between bilateral M1 while excitatory rTMS decreased it. This previous study employed a region of interest-based analysis in the stimulated region in M1, and suggests that interhemispheric functional connectivity can be utilized to examine the spatial extent of cortical plasticity in the stimulated region by calculating the connectivity in a voxel-by-voxel basis, with no assumption of functional symmetricity of the cortex.

In the present study, to examine the spatial extent of cortical plasticity, we measured interhemispheric functional connectivity changes in the first dorsal interosseous (FDI) representation in the M1 in the left hemisphere. Quadripulse stimulation (QPS) [33, 40, 45, 46] was applied to induce changes in cortical excitability in the M1, while the position and orientation of the stimulation coil were constantly monitored by an online navigator. The voxel-wise changes of interhemispheric functional connectivity after QPS were calculated to visualize the spatial extent of cortical plasticity. Localizer scans of the finger movement task were also administered to compare the spatial extent of brain activation in the M1 with that of changes in interhemispheric functional connectivity.

## Materials and methods

### Subjects

Twenty right-handed subjects [12 men and 8 women, age: 25.9 ± 9.0 years (mean ± SD) ranging from 20 to 48 years] participated in the experiments. Written informed consent was obtained from all subjects according to the Declaration of Helsinki. The experimental procedures were approved by the Institutional Review Board of Juntendo University School of Medicine.

### Overall design of the combined rTMS-fMRI experiment

**Fig 1:**
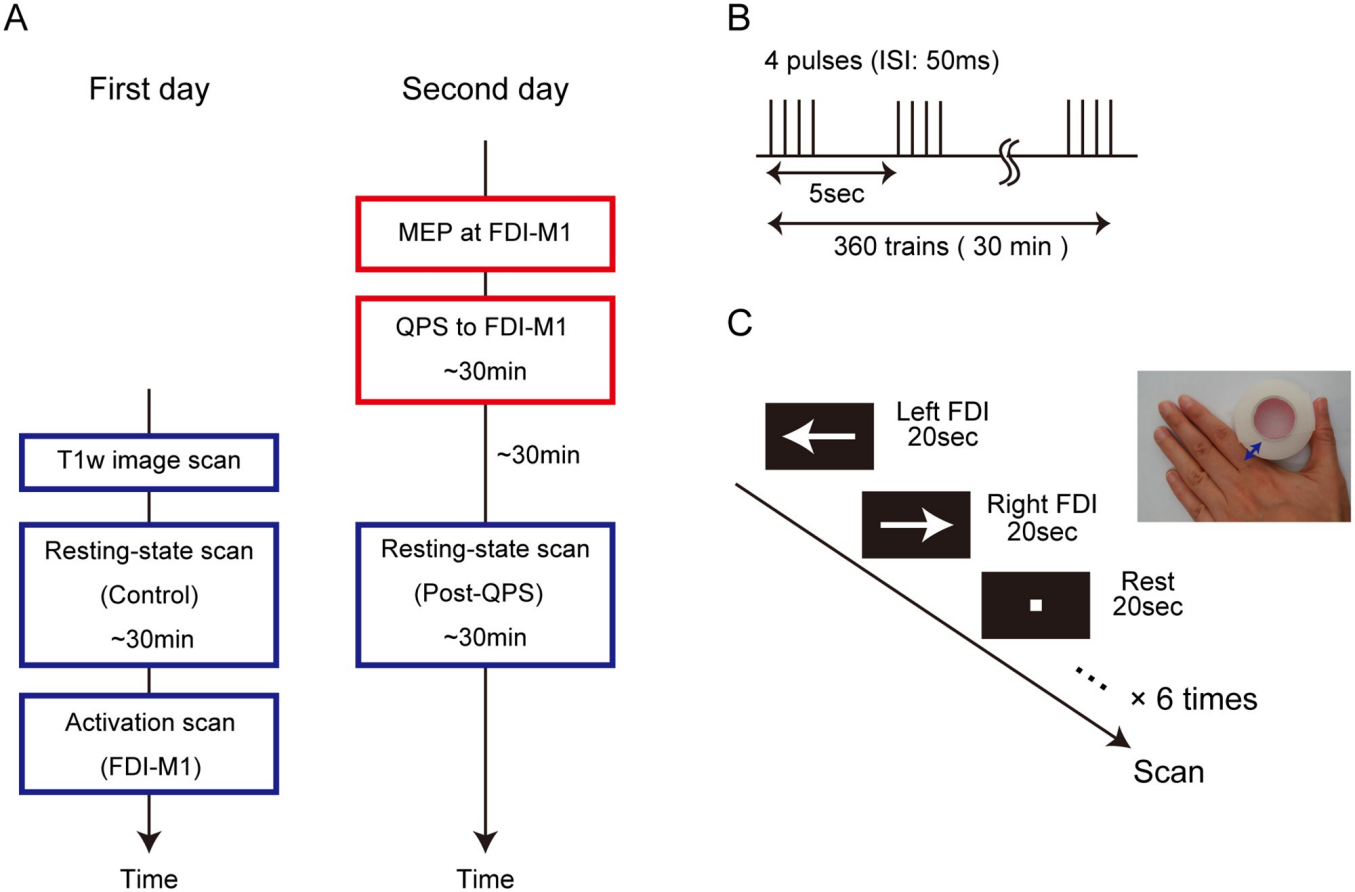
Overview of the experimental design. (A) On the first day, the T1-weighted image and resting-state (control) scans were obtained. On the second day, MEP after single-pulse TMS was measured to identify the FDI representation in the M1. Then, QPS was delivered to the FDI-M1 region for 30 min to induce cortical plasticity. After an approximately 30 min break, the resting-state scan (post-QPS) was administered. (B) A QPS sequence that consisted of 360 trains of quadripulse rTMS at 50 msec inter-stimulus interval (ISI) with an inter-train interval of 5 sec. (C) A finger movement task in a localizer scan to identify the M1 for the FDI. The subjects were instructed to move their left or right FDI at 2Hz as the arrows blinked for 20 sec each, followed by resting for 20 sec. The left-right-rest cycle was repeated six times.

The experiment consisted of two daily sessions (Fig 1A). On the first day, T1-weighted structural images were acquired. Then, the subjects underwent a resting-state scan for five runs without QPS as a control connectivity scan. On the second day, the motor evoked potential (MEP) was measured to search for the hot spot of the FDI representation in the M1. Then, QPS was delivered to the hot spot for 30 min to induce cortical plasticity at the FDI-M1. An online navigation system was utilized to maintain accurate stimulation onto the hot spot throughout the 30 min of QPS. After an approximately 30 min break (during which the subject was moved from a TMS room to an MRI scanner, placed into the scanner and administered with preparatory scans), a resting-state functional scan was administered for five runs to measure functional connectivity changes induced by QPS. A functional localizer scan was also administered for one run, where the subjects performed a motor task designed to activate the FDI-M1.

### TMS procedures

TMS was administered using a hand-held figure-of-eight coil (7-cm diameter at each wing; The Magstim, Whitland, Dyfed, UK). Single-pulse TMS experiments were conducted to determine the optimal stimulation site and active motor threshold (AMT) for the right FDI muscle [33, 40, 45–48]. MEP was recorded from the right FDI muscle using Ag/AgCl sheet electrodes placed over the muscle belly (active) and the metacarpophalangeal joint of the index finger (reference). The signals were sent to an amplifier (MEG-5200, Nihon Kohden, Japan) through filters set at 150 Hz to 3 kHz. The AMT was defined as the lowest intensity that evoked a small response (>100 μV) in more than 5 of 10 consecutive trials when the subjects maintained a slight contraction of the right FDI (10% of the maximum voluntary contraction [MVC]) [33, 45–47]. MVC was calculated approximately 10 min before QPS administration.

There are many forms of rTMS that are widely used to induce neural plasticity [4, 49] including theta burst stimulation (TBS) [50], high-frequency rTMS [51] and low-frequency rTMS [52]. As the fMRI measurements took more than one hour (including moving from the TMS room to the MRI scanner, placing the subject into the scanner, performing preparatory scans, and resting-state measurements), an rTMS paradigm with aftereffects lasting over an hour was needed. QPS, the effect of which lasts approximately 90 min [45, 46], offered a suitable length of aftereffect for the present study.

Magnetic pulses of QPS were delivered by four magnetic stimulators (Magstim 200^2^, The Magstim) connected to a specially designed combining module (The Magstim). QPS consisted of trains of four monophasic TMS pulses with an inter-train interval (ITI) of 5 sec, based on the standard protocol of QPS [45, 46, 53] (Fig 1B). Each train consisted of four magnetic pulses separated by inter-stimulus intervals (ISIs) of 50 msec (inhibitory QPS). One QPS block consisted of 360 consecutive trains that took 30 min. The intensity of QPS was set at 90% AMT and was 40.8 ± 7.0 (mean ± SD) % of the maximum stimulator output. We did not use QPS with an ISI of 5 msec (excitatory QPS) because it is well known that facilitation is often associated with surround inhibition [54, 55]. It is well established that the inhibitory QPS reduces MEP by approximately 50% for approximately 90 min [45, 46]. The QPS effect in MEP was also confirmed in our previous study of fMRI-rTMS [33]. Furthermore, the magnitude of connectivity changes has been shown to correlate with changes in MEP [38]. Based on these literatures, we did not record MEP to confirm the effect of QPS in this study. After QPS administration, the subjects were asked if they had a headache or any other type of discomfort. No subjects reported any discomfort.

An online navigator assured that stimulation was targeted to the left FDI-M1 determined by the MEP measurements. T1-weighted images were registered to subjects’ heads in space using a tracking device and navigator software (TMS Navigator-SW, Localite GmbH, Germany). The position and orientation of the coil were also registered to the subjects’ heads in space and were continuously monitored and recorded in real time during QPS.

### fMRI procedures

Image data were acquired using a 3-T MRI scanner and a 64-channel RF head coil (Siemens Prisma, Erlangen, Germany). T1-weighted structural images were obtained for anatomical reference (resolution = 0.8 × 0.8 × 0.8 mm^3^). Functional images were obtained using multi-band gradient-echo echo-planar sequences [56] (TR = 1.0 sec, TE = 30 msec, flip angle = 62 deg, FOV = 192 × 192 mm^2^, matrix size = 96 × 96, 78 contiguous slices, voxel size = 2.0 × 2.0 × 2.0 mm^3^, multi-band factor = 6, phase encode direction: posterior to anterior). Before each run, one functional image was acquired with opposite phase-encode direction for subsequent topup distortion correction [57].

The resting-state fMRI scan consisted of five runs of 6 min each, and the subjects were instructed to fixate on a cross during the scans. The localizer scan was also conducted to identify the M1 for the right FDI and consisted of one run of 6 min. During the FDI motor task, a left or right arrow appeared and blinked in the display, and the subjects were instructed to move their left or right FDI at 2 Hz as the arrows blinked for 20 sec each, followed by resting for 20 sec (Fig 1C). The left-right-rest cycle was repeated six times during the run.

### Image analysis for resting-state data

Images were first slice timing corrected, realigned using SPM8 (www.fil.ion.ucl.ac.uk/spm/), and topup distortion corrected using FSL [58]. For topup distortion correction, the susceptibility-induced off-resonance field was estimated using images with distortions going in opposite directions [57]. Temporal filters (0.009 Hz < f < 0.08 Hz) were applied to images using in-house-written Matlab scripts. A general linear model (GLM) [59] was used to regress out nuisance signals that correlated with head motion, whole-brain global signal, averaged ventricular signal, and averaged white matter signal. To prepare for subsequent interhemispheric functional connectivity analyses, obtained residual images were made symmetrical by spatial normalization to the MNI template and were spatially smoothed [full width at half maximum (FWHM) = 4 mm].

**Fig 2:**
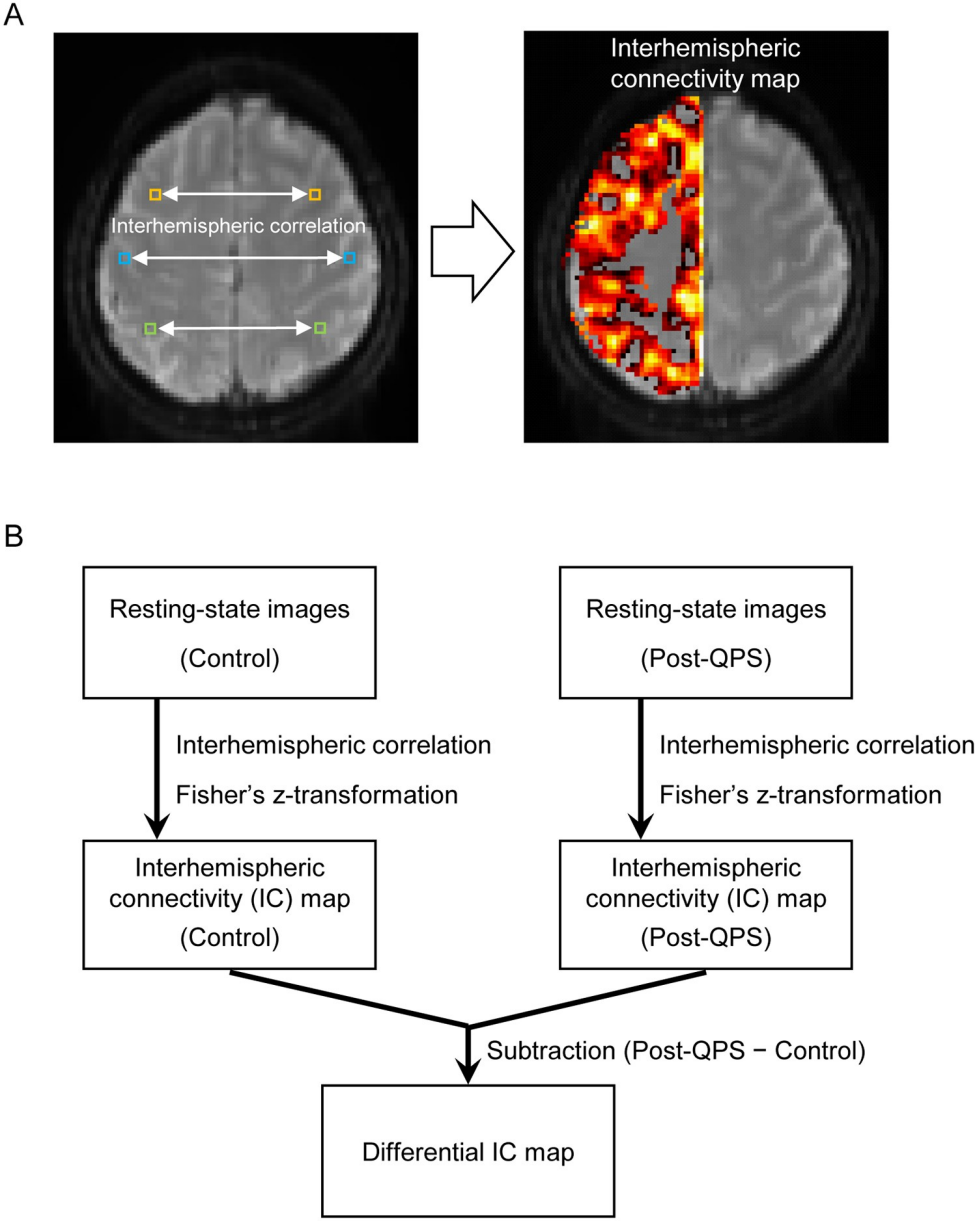
Interhemispheric functional connectivity analysis. (A) Each voxel in the left hemisphere of each subject was used as a seed to calculate its interhemispheric correlation with the corresponding voxel in the right hemisphere. The z values are shown only in the left hemisphere for display purposes. (B) The interhemispheric connectivity map of the post-QPS scans was calculated and was contrasted with that of the control scans to generate the differential interhemispheric connectivity map of each subject.

Then, we estimated how QPS changed the voxel-wise inter-hemispheric functional connectivity (Fig 2A). Each voxel in the left hemisphere of each subject was used as a seed to calculate its correlation with the corresponding voxel in the right hemisphere. For the corresponding voxel in the right hemisphere, the X coordinate of the voxels in the left hemisphere was flipped. A voxel-wise interhemispheric correlation was calculated for each seed voxel, and the correlation coefficient was then converted to Fisher’s z [60, 61] (Fig 2B). Since the interhemispheric connectivity map is symmetrical by definition, the z values are shown only in the left hemisphere for display purposes (Fig 2A). The z-map of the post-QPS was then contrasted with that of the control scans in each subject (Fig 2B). The differential interhemispheric connectivity map was transformed back into the original space for individual analyses. For group analyses, the spatial smoothing kernel was greater (FWHM = 6 mm) than that for single subject analyses (FWHM = 4 mm), and the differential z-maps were entered into a second-level one-sample t-test, treating subjects as a random effect.

### Image analysis for localizer scan data

Similarly to the analysis for resting-state data, images were first slice timing corrected, realigned, and distortion corrected using topup. The images were then spatially smoothed (FWHM = 4 mm). Time-series data were analyzed with a block design. The event timings of two types of trials (moving right/left FDI) were coded into a GLM, together with temporal and dispersion derivatives using the canonical hemodynamic response function. Six parameters of head motion derived from realignment were also included in the model as covariates of no interest. The right FDI-M1 in the left hemisphere was determined by calculating contrast images defined as moving right FDI > moving left FDI for each subject, to counterbalance non-motor components such as visual response to a cue and effort of finger movements. For group analyses, images were normalized to the template and were spatially smoothed with a greater kernel (FWHM = 6 mm), and contrast images were entered into a second-level one-sample t-test, treating subjects as a random effect.

## Results

### Stability of the stimulation site

**Fig 3:**
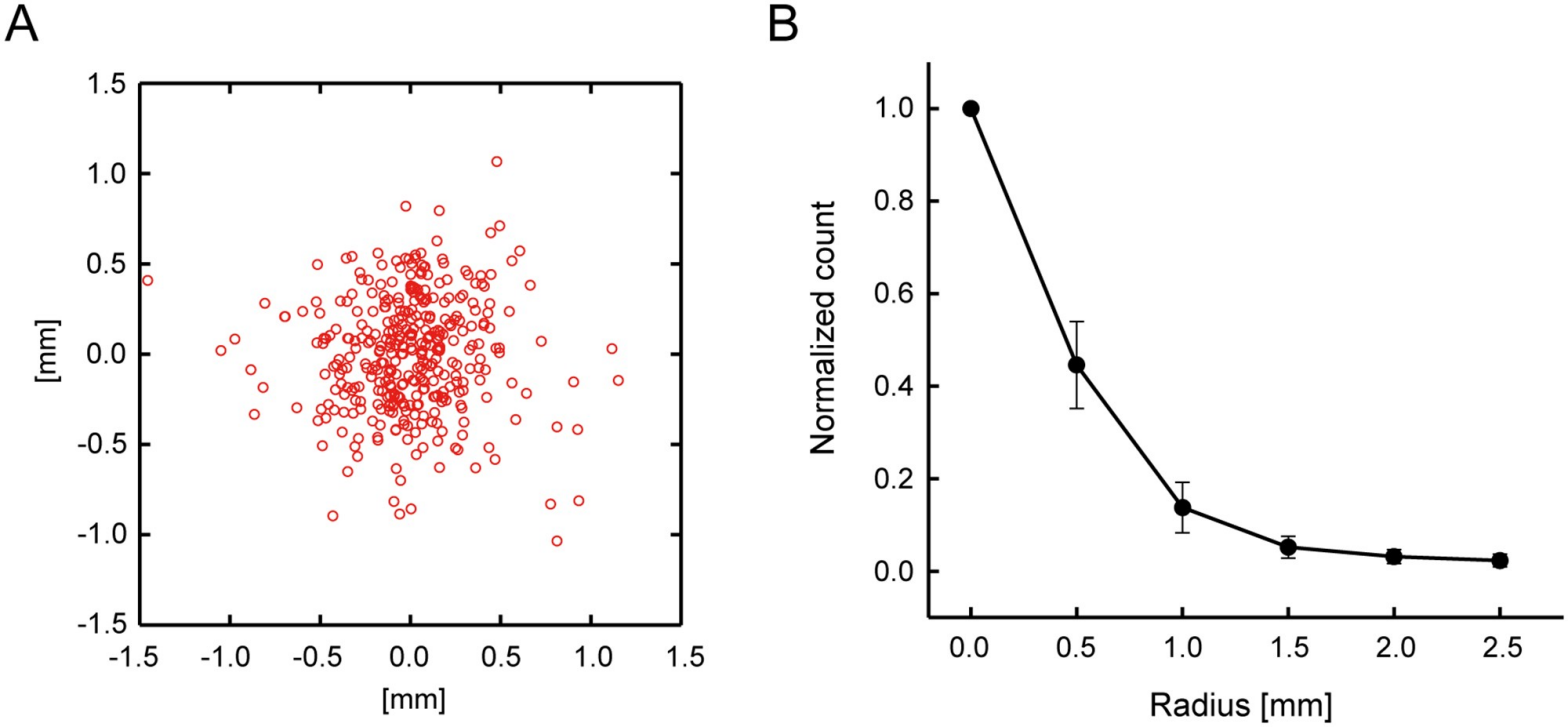
Monitoring of the stimulation site during QPS using an online navigation system. (A) The points of stimulation on the plane contacting the brain surface in one representative subject. Each dot represents one position of one-train stimulation. (B) The distribution of the stimulation points in the subject group as a function of the distance from the center. The counts with distances between 0 to 0.25 mm from the center were normalized to 1. The error bars indicate the standard error of means (SEM) of the subject group.

To maintain spatially accurate stimulation throughout the 30-min QPS, the online navigation system was utilized. The variability of the points of stimulation was estimated to confirm the spatial extent of the stimulation site. Fig 3A shows the points of stimulation on the plane contacting the brain surface in one representative subject. One count represents the coil position during one train of four pulses. The distribution of the stimulation points in the subject group is shown in Fig 3B as a function of the distance from the center. Counts with distances between 0 to 0.25 mm from the center were normalized to 1. The half width at half maximum (HWHM), which is a half of FWHM and measures the cluster extent from its center, of the counts was approximately 0.5 mm. Most of the stimulation points (96.0 ± 6.0%, mean ± SD) were located within a circle of 1 mm radius. The distance between the coil and the brain surface is known to be approximately 15 mm [62–65], and the variability increases as the stimulation goes deeper into the brain. However, navigation monitoring results confirmed that the stimulation site was reasonably stable compared to the size of a cluster of connectivity changes described later.

### Changes in interhemispheric functional connectivity after QPS

**Fig 4:**
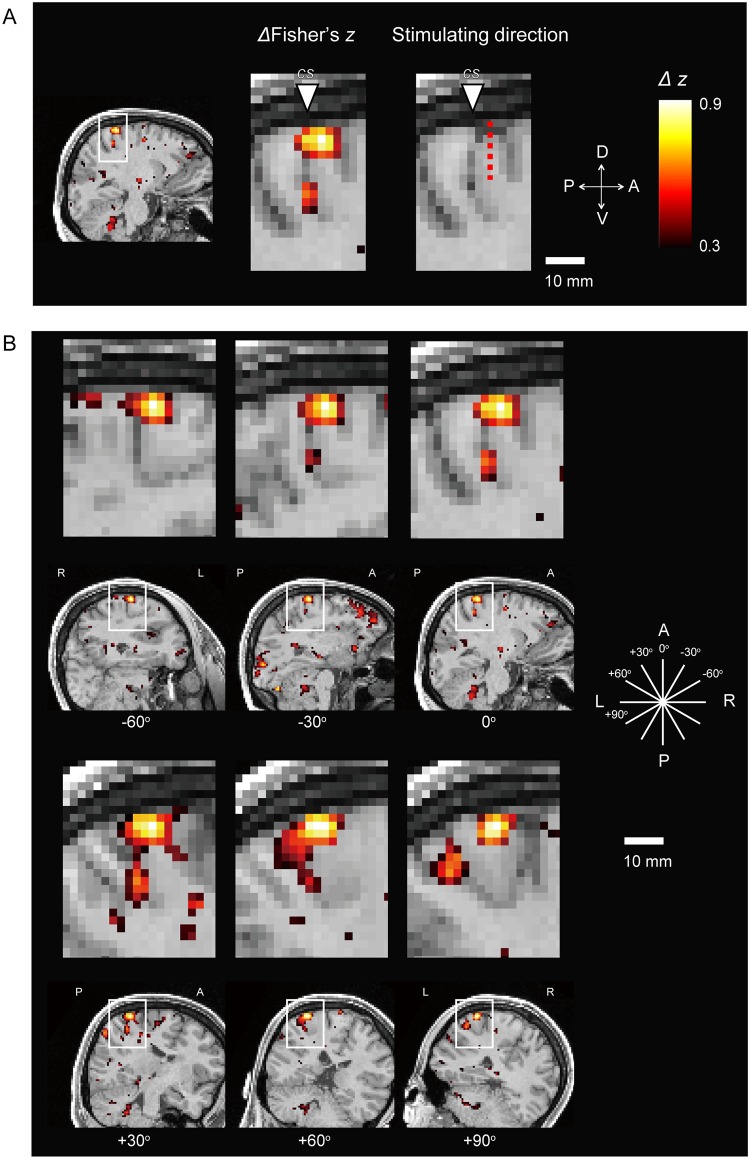
Differential interhemispheric connectivity map. (A) The differential interhemispheric connectivity map overlaid onto a structural image in one representative subject. The triangle indicates the central sulcus in the left hemisphere, and the red dashed line indicates the stimulation vector (the length of the vector in the figure is arbitrary). The color scale represents the differential Fisher’s z value. D: dorsal, V: ventral, A: anterior, P: posterior. (B) The differential interhemispheric connectivity maps sliced by different angles around the stimulation vector. L: left, R: right.

We estimated changes in cortical plasticity by calculating the difference in interhemispheric functional connectivity between the post-QPS and control scans. Fig 4A demonstrates a cluster of voxels with changes in the interhemispheric functional connectivity observed in the central sulcus in one representative subject. The cluster was spatially restricted around the stimulation site in the central sulcus, extending to the brain along the stimulation vector. The stimulation vector was perpendicular to the cortex as long as the experimenter stimulated the region indicated by the navigator system. Fig 4B shows the spatial extent of the cluster when the differential connectivity maps were sliced by different angles around the stimulation vector. The cluster of high connectivity changes appeared similar in spatial extent, irrespective of the different angles.

**Fig 5:**
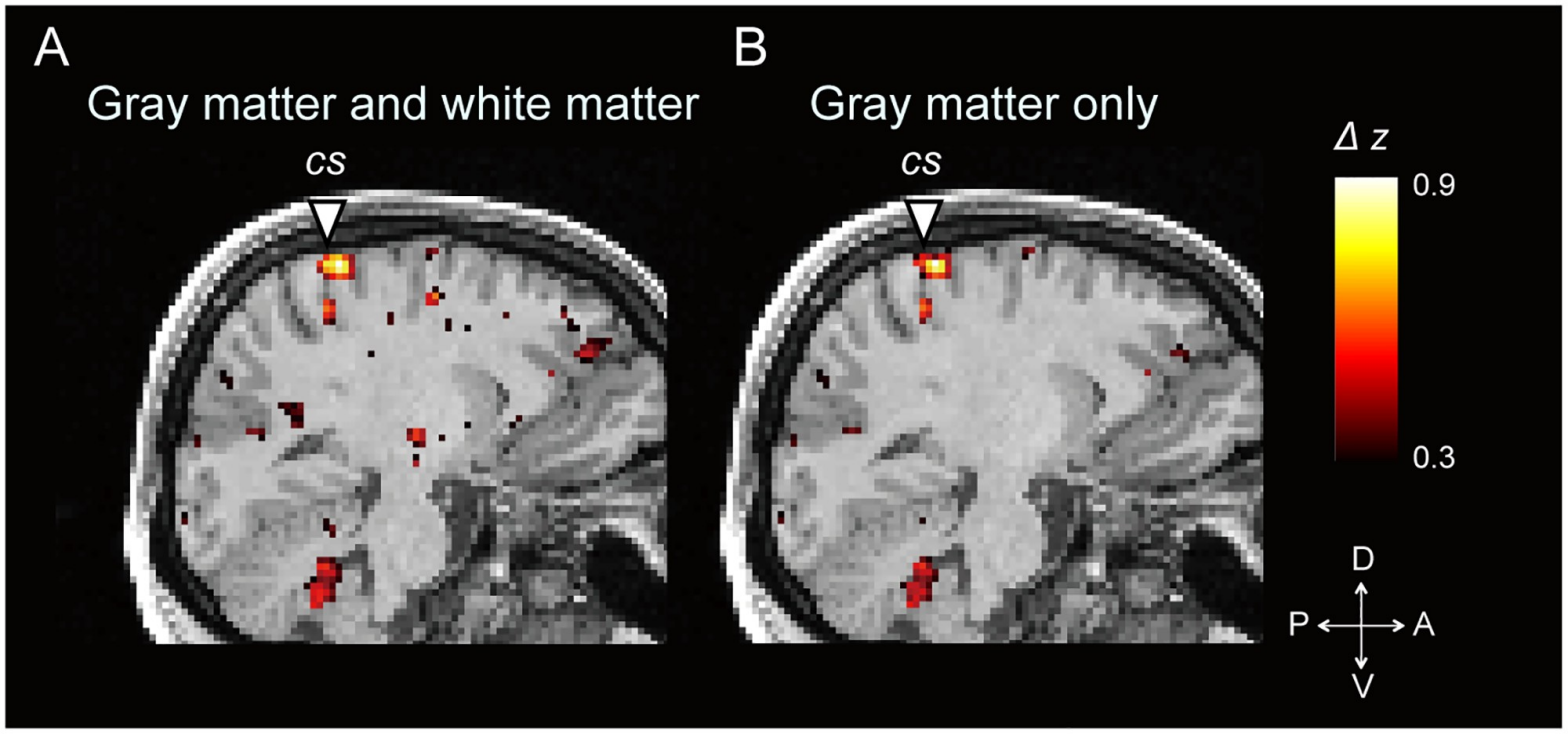
Two ways of calculating interhemispheric connectivity maps. (A) A differential interhemispheric connectivity map calculated simply between symmetrical voxels in the two hemispheres. Therefore, the voxel in the contralateral hemisphere can be gray matter or white matter. The map is the same as Fig 4A. (B) A differential interhemispheric connectivity map calculated in another way. The map was calculated between a gray matter voxel in one hemisphere and the gray matter voxel located nearest to the corresponding voxel in the contralateral hemisphere, if the corresponding voxel is judged as white matter.

Interhemispheric functional connectivity was calculated based on the assumption that when a voxel in one hemisphere is gray matter, a voxel in the contralateral hemisphere is also gray matter. However, this is not always the case. To address this issue, interhemispheric functional connectivity was calculated between a gray matter voxel in one hemisphere and the gray matter voxel located nearest to the corresponding voxel in the contralateral hemisphere, if the corresponding voxel is judged as white matter based on the segmentation process in SPM. Fig 5 shows differential interhemispheric connectivity maps calculated in these two ways. The spatial patterns of the z values were almost the same in the central sulcus, as well as in other clusters of no interest outside the central sulcus. The results validate the differential interhemispheric connectivity pattern calculated simply between symmetrical voxels.

**Fig 6:**
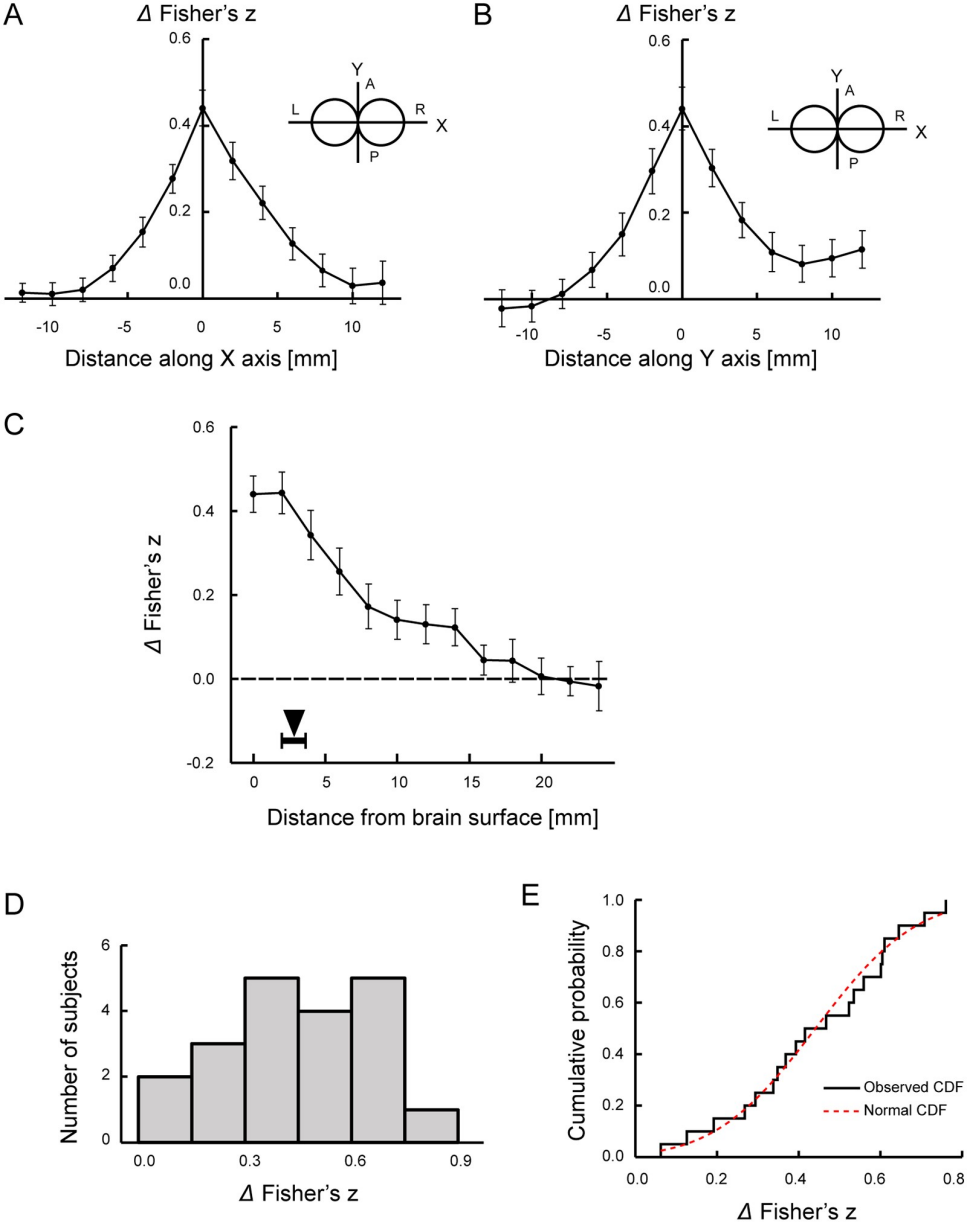
Cluster of connectivity changes in the surface and depth directions. (A) The differential Fisher’s z plotted along the long axis (X) in the brain surface. Vertical error bars indicate the SEM of the z values. (B) The differential Fisher’s z plotted along the short axis (Y) in the brain surface. (C) The differential Fisher’s z plotted along the stimulation vector (Z) from the brain surface. Data in white matter voxels were excluded from group averaging. The black triangle indicates the mean depth of the activation peak in M1, and the horizontal error bar indicates the SEM of the depth of the activation peaks. (D) Distribution of differential Fisher’s z in the subjects at the origin (X = 0, Y = 0, Z = 0) of the connectivity cluster. (E) Cumulative distribution function of the differential Fisher’s z of the subjects (shown in black). A red curve indicates the case of a normal distribution.

We then estimated how far the connectivity cluster extended in the brain surface and along the stimulation vector. Fig 6A and 6B show the differential Fisher’s z of the connectivity cluster in the brain surface along the long (X) and short (Y) axes of the stimulation coil, respectively. The cluster was spatially extended approximately 10 mm from the stimulation site (HWHM: approximately 3 mm), to a significantly greater extent in the anterior than in the posterior direction along the Y axis [t(19) = 2.2, P < 0.05], reflecting the direction of magnetic field from the stimulation coil. In Fig 6C, the differential Fisher’s z was plotted along the stimulation vector from the brain surface. Data in white matter voxels were excluded from group averaging. The z value gradually declined along the vector up to approximately 20 mm in depth (HWHM: approximately 7 mm). For reference, the distance from the coil and brain surface was 16.1 ± 2.9 mm (mean ± SD). Fig 6D shows the inter-individual variability of the differential interhemispheric connectivity at the origin of the cluster (X = 0, Y = 0, Z = 0). There were no subjects with a negative z value. The distribution was normal (Kormogorov-Smirnov test, P > 0.9) (Fig 6E). These results suggest that some subjects were less sensitive but were within a normal distribution.

To examine the effect of stimulation strength (i.e., 90% AMT) on differential interhemispheric functional connectivity, correlation was calculated between the differential z score and the stimulation strength across subjects. There was no significant correlation (r = -0.1, P > 0.05), suggesting that stronger stimulation does not result in greater connectivity changes.

### Brain activation during FDI movement

**Fig 7:**
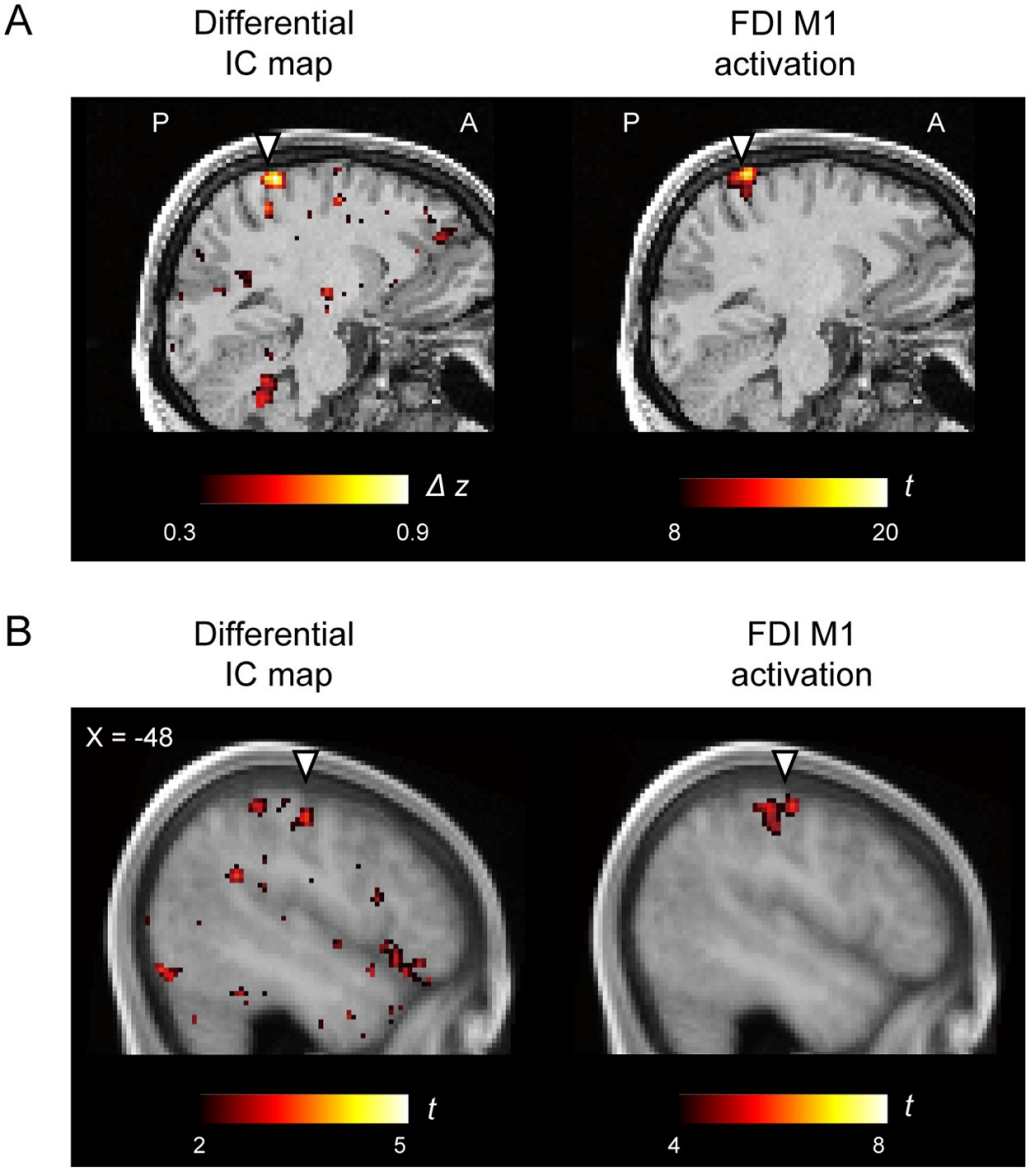
Comparison of differential interhemispheric connectivity and brain activity during finger movement. (A) The differential interhemispheric connectivity map (left) and brain activation map (right) in one representative subject (the same as Figs 4 and 5) in the original subject space. Triangles indicate the central sulcus of the subject. The color scale represents the differential Fisher’s z value (left) or t-value (right). (B) Group results of the two maps in MNI space. Triangles indicate the central sulcus.

To validate differential interhemispheric connectivity maps, brain activity was measured using fMRI while the same subjects performed a motor task designed to activate the right FDI representation in the M1 in the left hemisphere. The interhemispheric connectivity difference should be greatest near the surface (Fig 6), while the brain activation peak may be not always located near the surface. Therefore, the peaks of the interhemispheric connectivity difference and brain activation will not always overlap, but the clusters of the two should spatially overlap. Fig 7A shows the differential interhemispheric connectivity map and brain activation map in one representative subject in the original subject space (see also S1 Fig). The peaks of the two maps did not overlap, but their clusters considerably overlapped, especially in the central sulcus region stimulated by QPS. Fig 7B shows the group results of the two maps in MNI space (see also S1 Fig). These two maps also exhibited considerably overlapping patterns, confirming that the largest part of the cluster of connectivity changes in the central sulcus is located at the right FDI representation in the M1.

## Discussion

The present fMRI study utilized interhemispheric functional connectivity to examine the spatial extent of cortical plasticity induced in M1 by applying QPS with good spatial accuracy supported by an online navigator. A cluster of connectivity changes was observed mostly in the restricted region in the central sulcus, around a circle of 20 mm in diameter. The cluster extended in depth by approximately 20 mm. The activation related to finger movement in the left central sulcus region overlapped with the cluster of connectivity changes. These results indicate that connectivity changes in M1 were relatively restricted in space and suggest that interhemispheric functional connectivity can be used for visualization of cortical plasticity induced in the stimulated region.

Changes in interhemispheric connectivity after QPS to the left M1 were rarely seen outside the M1. Interhemispheric connectivity changes in the M1 indicate changes in connectivity between the left M1 (stimulated) and the right M1. Stimulation to the left M1 may induce changes in connectivity between the left M1 and the ipsilateral regions such as the premotor cortex. On the other hand, interhemispheric connectivity changes in the premotor cortex indicate changes in connectivity between the left premotor cortex and the right premotor cortex. Therefore, the interhemispheric connectivity changes outside the M1 require multi-step connectivity changes, which may explain the faint interhemispheric connectivity changes outside the M1.

It must be noted that cortical plasticity estimated using interhemispheric connectivity has potential limitations. First, the stimulation of one region may lead to changes in cortical excitability in other regions, as well as deeper parts of the stimulated region, in the same brain network [22, 27, 40, 43]. Although we only observed faint effects outside the M1, interhemispheric connectivity changes may detect plasticity induced outside of the stimulated region. It is also possible that the connectivity changes in the deeper part of the stimulated region may have been induced indirectly. Second, connectivity in the stimulated region may not always change after intervention. For example, the interhemispheric connectivity did not change after 1-Hz stimulation to the inferior parietal lobule (Fig 2 in Eldaief et al., 2011 [22]). Although we have shown that QPS affected interhemispheric functional connectivity in our present and previous [33] studies, it is unclear how generally the interhemispheric connectivity can be changed in various forms of non-invasive brain stimulation. Third, structural and functional asymmetry between the left and right hemispheres exists in some brain regions, and a high degree of interhemispheric asymmetry may hinder the application of our analyses. Although it is difficult to validate the visualization in all brain regions, the present study may present one successful case in M1 with validation of visualization of cortical plasticity using brain activation during finger movement.

It is known that fatiguing muscles immediately before rTMS can evoke changes in neural activation. For example, the reductions in MEP caused by continuous TBS (cTBS) are abolished if cTBS is performed after a 2-min period of MVC [66, 67]. However, a 1-min period of MVC has been shown to not cause any lasting MEP changes [68]. In the present study, MVC was calculated approximately 10 min before QPS administration and lasted approximately only 3 sec. Therefore, the effect of MVC in this study, if any, would be excitatory and would not explain the connectivity changes induced by inhibitory QPS that we observed. However, one potential limitation would be that repeated stimulation on M1 can create lasting tingling sensations. As no control comparison was done for the sensation, it is unclear whether cutaneous changes caused by the repeated pulses affected the functional connectivity changes seen in this study.

Previous studies have estimated the electric field elicited by TMS that decays as a function of the distance from the TMS coil [5–7, 10]. The average distance between the TMS coil and the brain surface is approximately 15 mm, both in the present study and in previous studies [62–65], and the average depth of the cluster of connectivity changes was approximately 20 mm in the present study. Based on the data from previous studies on electric field measurements [5–7], the strength of the electric field in the bottom of the cluster (i.e., approximately 35 mm away from the TMS coil) decays approximately by 60 to 70% from the brain surface (i.e., approximately 15 mm away from the TMS coil), suggesting that connectivity changes can be induced by at least 30 to 40% of the electric field strength at the brain surface. The previous data of electric field measurements also suggest that connectivity changes should extend approximately 100 mm in the brain surface, where the electric field strength is almost equivalent to that at 20 mm below the center of the brain surface [6, 7]. However, connectivity changes in the gyral surface were almost restricted to 10 mm in radius in the present study, presumably because the neurons in the gyral surface are relatively less sensitive to stimulation due to the under-optimal direction of the cortical layer relative to the TMS coil [8–11].

A previous study of electroencephalography applying rTMS to the M1 reported the spatial distribution of potentiation of cortical evoked potentials outside the M1, primarily in the bilateral premotor cortex [69]. The present study examined cortical plasticity at the stimulated region itself, the M1. It is critical to identify the spatial extent of intervention to investigate the brain-behavior relationship [70]. Visualization of the spatial extent of experimental intervention is commonly employed in animal studies, such as histological inspection of electrolytic marking for electrophysiological stimulation/recording [71–74] and intracortical virus injection for optogenetics/chemogenetics [75, 76], and visualization of intracortical drug injection using an MRI contrast agent [77, 78]. Moreover, recent advances in analyses of resting-state functional connectivity have allowed us to parcellate brain structures into numerous small functional regions [48, 79–94], highlighting the importance of accurate spatial estimation of the intervention site. The present study provides a potential way to visualize the spatial extent of intervention by rTMS in human subjects.

## Supporting information

S1 Fig Brain activation maps for a single subject and group subjects. (A) A brain activation map in one representative subject (the same as Figs 4, 5 and 7) shown in a transverse section of MNI space. Triangles indicate the central sulcus of the subject. The color scale represents t-value. The activation was present over the precentral hand knob. (B) A brain activation map of the group result.
(TIF)
