# Supplementary figures and images for: MRI-based visualization of rTMS-induced cortical plasticity in the primary motor cortex
Source: PLoS One. 2019 Oct 24;14(10):e0224175. doi: 10.1371/journal.pone.0224175 (PMC6812785; doi:10.1371/journal.pone.0224175)

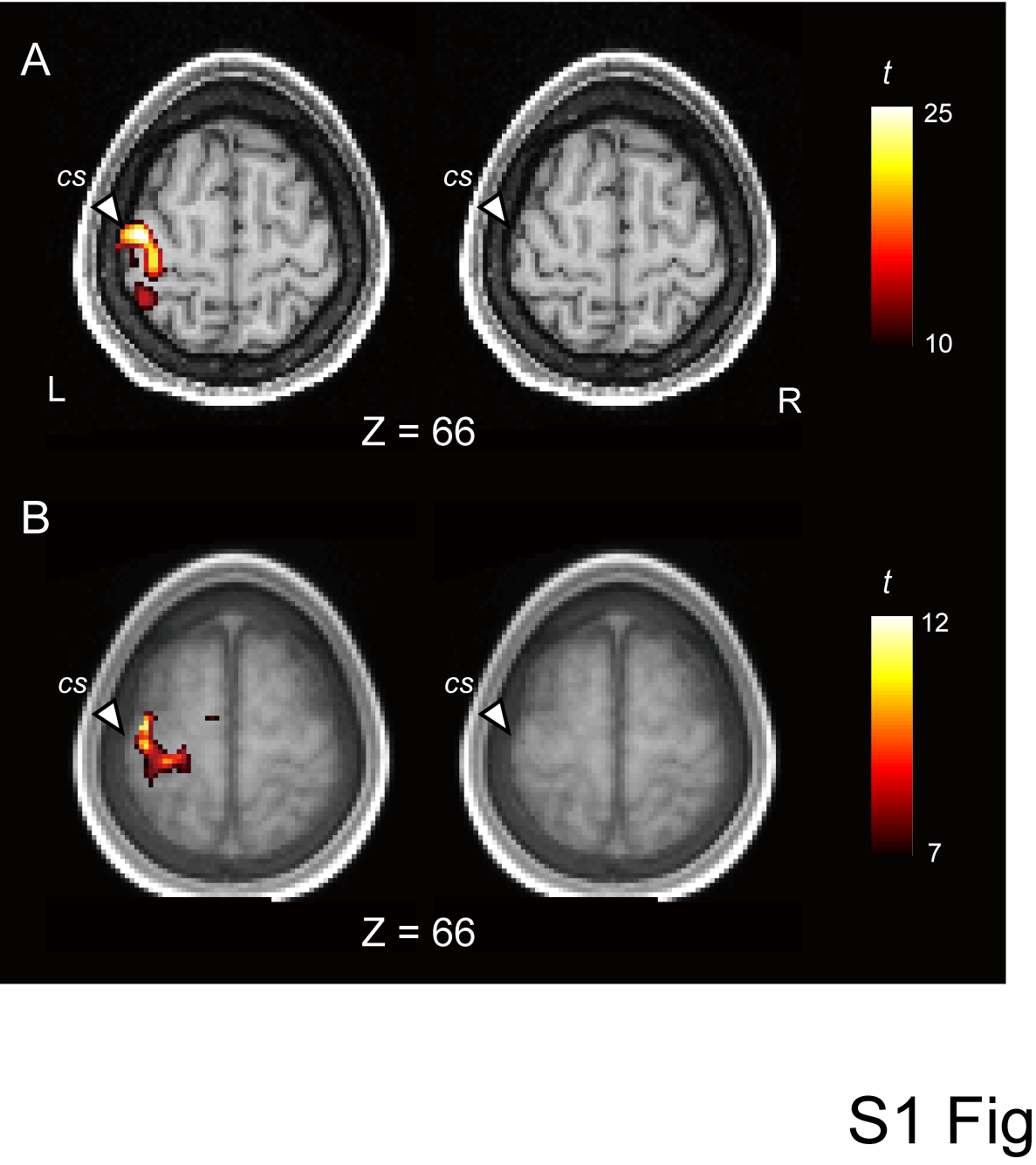

Supplement: S1 Fig — (A) A brain activation map in one representative subject (the same as Figs 4, 5 and 7) shown in a transverse section of MNI space. Triangles indicate the central sulcus of the subject. The color scale represents t-value. The activation was present over the precentral hand knob. (B) A brain activation map of the group result. (TIF) [file pone.0224175.s001.tif]
